# Behavioral Inhibition/Activation Systems and Depression Among Females With Substance Use Disorder: The Mediating Role of Intolerance of Uncertainty and Anhedonia

**DOI:** 10.3389/fpsyt.2021.644882

**Published:** 2021-03-04

**Authors:** Jinlan Xie, Ping Fang, Zhihao Zhang, Ronglei Luo, Bibing Dai

**Affiliations:** ^1^Department of Psychology, Capital Normal University, Beijing, China; ^2^Department of Psychiatry and Psychology, School of Basic Medical Sciences, Tianjin Medical University, Tianjin, China; ^3^Xiangya Nursing School, Central South University, Changsha, China

**Keywords:** behavioral inhibition/activation systems, intolerance of uncertainty, anhedonia, depression, females with substance use disorder

## Abstract

Previous studies have shown that the behavioral inhibition/activation systems (BIS/BAS) have substantial effects on substance use disorder and emotional disorders, and substance use disorder and emotional disorders often occur; in particular, females with substance use disorder are more likely to also have serious emotional disorders including depression than their male counterparts. However, the associations between the BIS/BAS and depression in females with substance use disorder have received little attention. Furthermore, the underlying mechanisms of these relations are largely unknown. The present study examines the mediating roles of intolerance of uncertainty and anhedonia in the associations between the BIS/BAS and depression among females with substance use disorder from the Research Domain Criteria (RDoC) framework. A total of 303 females with substance use disorder from a compulsory substance abuse detention center were tested using a cross-sectional survey involving BIS/BAS Scales, Intolerance of Uncertainty Scale, Snaith-Hamilton Pleasure Scale, and Center for Epidemiologic Studies Depression Scale. The path analysis model revealed that both the BIS and BAS had a direct effect on depression, that the BIS had an indirect effect on depression through intolerance of uncertainty, and that the BAS had an indirect effect on depression via anhedonia. These findings contribute to a more thorough understanding of how the BIS/BAS influence depression among females with substance use disorder and suggest that the utility of targeting these associations in treatments would help reduce depression in females with substance use disorder.

## Introduction

Globally, substance use disorder (SUD) has been increasing rapidly over the past decade (1). In addition, SUD prevalences were generally greater for males compared to females at most ages until the 70s (2). For example, the prevalences of 12-month DSM-V SUD for males and females were 4.9 and 3.0% in the United States, respectively (3). Similarly, the prevalences of 12-month DSM-IV SUD for males and females were 3.6 and 0.3% in China, respectively (4). SUD can be defined as a chronic relapsing brain disease that urges patients with SUD to seek and compulsorily use substances, despite their significant adverse consequences (5), especially in patients with certain biological, psychological or physical vulnerabilities (6, 7). SUD can cause a range of acute and long-term negative consequences for individuals, such as hyperemesis syndrome, neurocognitive impairments, HIV infection, and premature death (1, 8). SUD are also associated with substantial societal costs from lost productivity, poverty, health care costs, violent and property crime (3). In addition, there is a close relationship between SUD and psychiatric comorbidities, especially, the high prevalence of the SUD-depression comorbidity (9, 10), and some common risk factors appear to lead both to SUD and psychiatric disorders (e.g., depression, anxiety, schizophrenia, bipolar disorder) (9, 11, 12). Furthermore, compared to suffering from only one disorder, suffering from comorbid SUD and depression is strongly correlated to more serious consequences (e.g., greater symptom severity, impairment, suicidality) (13). However, it is still unclear what common risk factors play an important role and how to influence both SUD and depression. In addition, women are likely to suffer from SUD faster when using substances occasionally and are more vulnerable to relapse than men (14). Furthermore, females with SUD are twice as likely as male with SUD to suffer from psychiatric disorders (e.g., depression, anxiety, schizophrenia, bipolar disorder), almost 30 vs. 16%, respectively (15). Thus, to promote the prevention and early intervention of depression, it is imperative to identify risk factors and underlying mechanisms for depression in females with SUD.

The National Institute of Mental Health (NIMH) recently launched the Research Domain Criteria (RDoC) initiative, which provides a new classification framework with transdiagnostic psychopathological dimensions for research on psychiatric disorders (16). These continuous dimensions which are important factors in the context of precision medicine for psychiatry, vary from the general population to individuals with psychiatric disorders (17). Furthermore, these transdiagnostic dimensions can be associated with a range of psychiatric disorders and be used to distinguish the different pathophysiological disease subtypes and serve as potential predictors of treatment outcomes (18). In addition, two objectives of the RDoC initiative are to explore the mechanisms common to a clustering of psychiatric disorders and the unique mechanisms corresponding to specific psychiatric symptoms serving as indicators of differential risk factors among these symptoms (19). Because depression is the first leading cause of global burden among psychiatric disorders (20) and there is high co-occurring SUD and depression in female (9, 15). Therefore, it is very important to explore the common and specific mechanisms for depression in females with SUD within the RDoC framework. To explore these mechanisms, we adopted the Interaction of Person-Affect-Cognition-Execution (I-PACE) theory (21) in the SUD field to build the present research model. The prior theoretical model suggests that potential predisposing variables and vulnerability factors, such as personality variables, cognition and affect-vulnerability factors, may moderately mediate the development and maintenance of psychiatric disorders.

Gray's neuropsychological reinforcement sensitivity theory postulates that two basic dimensions of motivation, including a behavioral inhibition system (BIS) and a behavioral activation system (BAS), govern avoidance and approach behaviors in response to various types of stimuli (11, 22). According to this theory, the BIS is sensitive to stimuli of punishment or non-reward, which may drive individuals to avoid potentially negative or harmful consequences. Therefore, individuals with high levels of BIS activation are more likely to avoid loss and to show a blunted response to reward (23). However, the BAS generates behaviors corresponding to all conditioned and unconditioned appetitive stimuli and displays close relationships with the enhancement of reward or the termination of punishment. Thus, individuals with high levels of BAS activation may show greater proneness to seek reward and to approach novelty (23). As transdiagnostic personality traits, the BIS and the BAS provide an important view for understanding and explaining psychopathology, such as anxiety disorders, depression, eating disorders, and SUD (11). Previous studies found that individuals with SUDs reported higher BAS levels than controls and that the BAS was positively associated with lifetime diagnoses of substance abuse without comorbid anxiety disorders (24, 25). However, the associations between the BIS and substance use problems are still inconsistent. Some studies have found a significant negative correlation between the BIS and substance use problems (26), while some studies have indicated that the BIS is not significantly associated with substance use problems (27). These inconsistencies may be caused by considering different kinds of substance use and different study populations (e.g., age groups, sex ratio) (28). In addition, a large amount of evidence supports the significant associations between the BIS/BAS and depression (11). Previous results indicated that low BAS sensitivity not only is a potential marker of vulnerability to depression but also may be useful in predicting the course of the disorder (29, 30). Furthermore, behavioral activation treatments aim to modify the pattern of low approach in depressed patients by positive activity scheduling and have played an important role in treating depressive episodes and reducing relapses (31). Although previous researchers initially considered the BIS a specific diathesis for anxiety rather than depression, many studies have recently indicated that BIS reactivity is positively related to depression (29, 32). For example, compared to a control group, a depressed group showed higher BIS levels. Furthermore, BIS scores have been shown to have a more positive association with depression scores in a major depressive disorder group than in a control group. Although the above results could indicate that Gray's neuropsychological reinforcement sensitivity theory may be useful for understanding and explaining psychiatric disorders, the inconsistent results on the association between SUD and depression require more research to explore the associations between the BIS/BAS and depression in a specific context, especially in females with SUD. Furthermore, although most previous studies have shown that the BIS/BAS contribute to depression, little is known about the mediating mechanisms underlying these associations in females with SUD. However, two important transdiagnostic psychopathological dimensions—intolerance of uncertainty as cognitive bias and anhedonia as emotion and motivation deficits—may be among the mechanisms for linking the BIS/BAS to depression in females with SUD.

Life is full of uncertainty, but the extent to which uncertainty is tolerable varies across individuals. Intolerance of uncertainty (IU) is a cognitive bias that influences individuals' perceptions, interpretations, and responses to uncertain scenarios at the cognitive, emotional, and behavioral levels (33). Individuals with a high level of IU experience stress and disturbance in response to uncertainty, hold a negative attitude toward uncertainty, believe uncertainty causes dysfunctional behavior, and regard uncertainty as unfair and to be avoided (34). Recently, IU has been explained as “an individual's dispositional incapacity to endure the aversive response triggered by the perceived absence of salient, key, or sufficient information, and sustained by the associated perception of uncertainty” (35). Although previous studies have suggested that IU plays an important and specific role in the development and maintenance of high levels of worry and generalized anxiety disorder (33, 36), an increasing number of researchers regard IU as a transdiagnostic risk factor for a range of psychiatric disorders, such as generalized anxiety disorder, obsessive-compulsive disorder, eating disorders, depression, and SUD (37–39). For example, several studies have found that compared to healthy individuals, individuals with SUD perceive higher levels of IU (12, 40). While the researchers who conducted these studies also suggested that although IU is a feature of SUD, it may not play a unique role. First, the BIS is related to attempts to escape from or avoid novel, threatening or uncertain environments, which may cause individuals to interpret ambiguous situations more negatively (41). Thus, individuals with high BIS levels may show enhanced associative learning and learn to avoid an aversive situation easily (42). Because both the BIS and IU are closely correlated with information processing biases about dangerous or ambiguous stimuli, the BIS seems to be an important predictor of IU (43). The BAS predisposes an individual to pursue reward and novel sources, while individuals with high levels of IU are likely to select low-probability immediate rewards rather than high-probability delayed rewards (44). Furthermore, compared to a control group, individuals with higher levels of IU were less sensitive to the previously rewarded context (40). Therefore, the BAS may have no association with IU. Second, individuals with higher IU tend to perceive uncertainty negatively and adopt negative coping strategies and poor problem orientations, which explains why IU can correlate positively with depression (45). For example, people diagnosed with depression have been shown to possess higher levels of IU than community and undergraduate samples (46). Furthermore, IU has been shown to predict not only current depressive symptoms but also depression levels 6 weeks later (47). Although previous studies have suggested that IU may act as a mediator between the BIS and depression, no study has explored these associations in females with SUD.

Traditionally, anhedonia has been conceptualized as the inability to experience pleasure or interest in things (5). Recently, anhedonia has been recognized as an important transdiagnostic psychopathological dimension according to the RDoC by the NIMH (48). Anhedonia is known as an important symptom across psychiatric diagnoses, including affective disorders, obsessive-compulsive disorder, schizophrenia and SUD (19, 49). In particular, anhedonia seems to play an important role in the pathogenesis of both SUD and mood disorders but is more likely to be associated with the cooccurrence of SUD and depression (9). The BAS has a strong relationship with reward seeking, and individuals with high BAS levels exhibit enhanced reward dependence and novelty processing (28). Previous studies have found low BAS levels and reduced motivation to pursue rewarding stimuli to be positively linked to anhedonia in a healthy population (50) and physical activity engagement to be negatively associated with anhedonia (51). Furthermore, Veldhoven, Roozen, and Vingerhoets (52) found that the BAS was negatively correlated with anhedonia in patients with an alcohol use disorder. A range of studies adopting multiple methods from self-report, behavioral and neurophysiological levels have provided strong evidence that reduced approach motivation and reward hyposensitivity reflect motivational deficits in anhedonia (52–54). However, individuals with high levels of BIS activation exhibit enhanced sensitivity toward punishment and threat stimuli, and they show a blunted responsivity to reward (23), which suggests that the BIS may not play an important role in anhedonia. In addition, cross-sectional and longitudinal studies have shown that anhedonia plays an important role in the development of depression (55, 56). Furthermore, anhedonia can predict poorer responsivity to pharmacological and psychological interventions for depression (57). Although previous studies have suggested that anhedonia is a potential mediator between the BAS and depression, the role of anhedonia in these associations has not been thoroughly studied in females with SUD.

**Figure 1:**
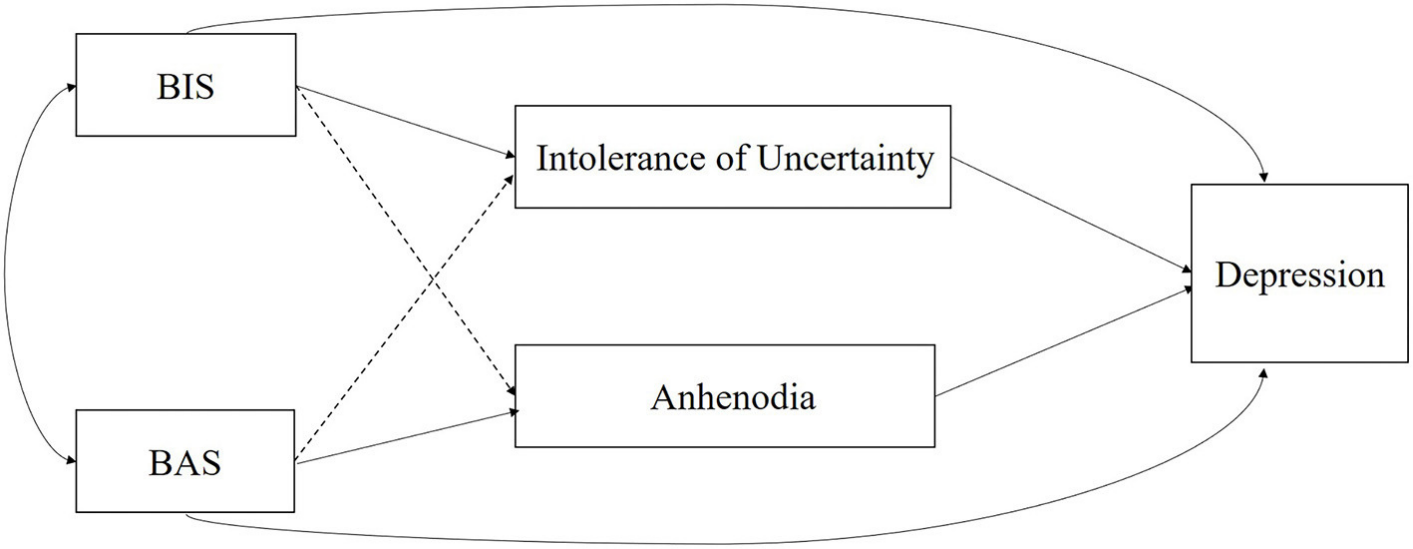
The supposed model.

The purpose of the present study was to examine how the BIS/BAS influence depression in females with SUD. Specifically, this study explored the mediating effects of IU and anhedonia on the BIS/BAS and depression. A previous study found that high BIS levels had an indirect effect on depression via increased rumination and that low BAS levels had an indirect effect on depression through decreased self-reflection in a sample of participants who had attempted suicide (58). To our knowledge, this is the first comprehensive empirical study incorporating the BIS/BAS, IU, anhedonia and their roles in depression among females with SUD within the RDoC framework. On the basis of the I-PACE theory, the proposed model is presented in Figure 1. It is plausible to hypothesize that IU and anhedonia act as mediators of the BIS/BAS and depression relationship. Therefore, our hypotheses are as follows: (1) There is a high prevalence of depression in females with SUD; (2) the BIS and BAS directly influence depression; (3) IU, rather than anhedonia mediates the relationship between the BIS and depression; and (4) anhedonia, rather than IU, mediates the relationship between BAS and depression.

## Materials and Methods

### Participants

Participants were recruited from a compulsory substance abuse detention center inTianjin, a Representative Municipality city of China. Inclusion criteria included: current diagnosis SUD, between the ages of 18–59, being sufficiently fluency in Chinese to complete the research questionnaires, and being willing to provide written informed consent prior to their inclusion. In addition, exclusion criteria comprised the following: traumatic brain injury and severe suicide risk. Based on the cluster sampling method, a total of 303 Chinese females with SUD were eligible. Of the participants (M_age_ = 34.97 years, SD = 8.52 years, age range: 18–57 years) included 41.9% were unmarried, 21.5% were married, 35.0% were divorced and 1.7% had missing marital status data. More detailed sociodemographic information is shown in Table 1. This cross-sectional design research was approved by the Institutional Review Board of Department of Psychology, Capital Normal University in China. All participants gave written informed consent and they could quit the study at any time without being penalized.

**Table 1:** Sociodemographic variables of participants (*N* = 303).

**Characteristic**	***n***	**%**
**Marital status**		
Unmarried	127	41.9
Married	65	21.5
Divorced	106	35.0
Missing	5	1.7
**Education**		
Primary school and below	67	22.1
Junior high school	145	47.9
High school	68	22.4
University and above	18	5.9
Missing	5	1.7
**Occupational status**		
Institutional personnel	4	1.3
Company employee	14	4.6
Freelancer	140	46.2
Other	137	45.2
Missing	8	2.6
**Duration of substance use (y)**		
1	17	5.6
2	36	11.9
3	32	10.6
4	37	12.2
5 or over 5	178	58.7
Missing	3	1
**Number of compulsory detoxification**		
1	142	46.9
2	106	35.0
3 or over 3	49	16.2
Missing	6	2
**Type of substance used**		
New substances (ecstasy, meth, etc.)	225	74.3
Traditional substance (heroin)	67	22.1
Missing	11	3.6

### Measures

#### The Behavioral Inhibition System/Behavioral Activation System Scales

The BIS/BAS scales are useful tools for studying individual differences in behavioral inhibition systems and behavioral activation systems (22). A validated Chinese version of the BIS/BAS scales was used to assess the BIS and BAS (59). The BIS/BAS scales comprise 18 items, including the BAS scale (13 items) and the BIS scale (5 items). The former scale is divided into three subscales: drive (BAS-drive, 4 items), reward responsiveness (BAS-reward, 5 items), and fun seeking (BAS-fun, 4 items). All items were assessed on a 4-point Likert scale from 1 (totally disagree) to 4 (totally agree). Sample items are “When I get something I want, I feel excited and energized (BAS)” and “I feel pretty worried or upset when I think or know somebody is angry at me (BIS).” In the present study, scores for all 13 BAS items were summed to yield a single BAS score, while scores for all five BIS items were added up to generate a single BIS score. Higher BAS and BIS scores reflect higher BAS and BIS levels, respectively. The Cronbach's alpha coefficients for the BAS and BIS in the current sample were 0.881 and 0.620, respectively.

#### The Intolerance of Uncertainty Scale

The IUS is widely used to assess individuals' extent of intolerance of uncertainty by rating 27 items on a scale from 1 (Not at all characteristic of me) to 5 (Entirely characteristic of me) (60). The IUS includes four subscales: uncertainty is stressful and upsetting (e.g., “Uncertainty makes life intolerable.”), uncertainty leads to the inability to act (e.g., “When it's time to act, uncertainty paralyzes me.”), uncertain events are negative and should be avoided (e.g., “One should always look ahead to avoid surprises.”), and uncertainty is unfair (e.g., “I think it's unfair that other people seem to be sure about their future.”) (34). The overall IU score is determined by summing all item scores, with higher scores indicating greater IU. The Chinese language version demonstrates excellent internal consistency, good test-retest reliability over a 5-week period, and adequate convergent and discriminant validity (61, 62). In the present study, the Cronbach's alpha coefficient for the Chinese IUS was 0.909.

#### The Snaith-Hamilton Pleasure Scale

The SHAPS is a self-administered scale including 14 items that is used to assess anhedonia (63). Each of the items has a set of four response categories: Strongly Agree (=1), Agree (=2), Disagree (=3), and Strongly Disagree (=4). A sample item is “I would enjoy being with my family or close friends.” Total scores range from 14 to 56, with higher scores indicating a higher level of anhedonia. The Chinese language version shows excellent internal consistency, good construct validity, and adequate convergent and discriminant validity (64). In the present study, the Cronbach's alpha coefficient for the Chinese SHAPS was 0.915.

#### The Center for Epidemiological Studies Depression Scale

The CES-D is used to assess depressive symptoms and includes 20 items in Likert format, using four possible responses anchored by 0 (rarely or none of the time) and 3 (most or all of the time) (65). Total scores range from 0 to 60, with higher scores indicating a higher level of depression. Respondents with scores equal to or >16 are defined as depressed (66). The CES-D has been extensively validated in Chinese populations (67). In the present study, the Cronbach's alpha coefficient for the Chinese CES-D was 0.878.

### Procedure

The participants were given a packet of questionnaires that included instructions on how to respond to the questions and assurances of anonymity as well as questions regarding their basic sociodemographic information (i.e., age, marital status, and education), the BIS/BAS scales, IUS, SHAPS, and CES-D. All scales were administered to the participants individually. All scales were printed in the Chinese language and took approximately 25 min to finish. No personal identifying information was collected, and all the information collected was confidential.

### Data Analysis

Because the proportion of data missing from each scale was low (<5%), mean substitution was used to deal with missing data. First, we conducted descriptive statistics and Pearson's correlation analysis with IBM SPSS statistics 24.0. Specifically, we analyzed the influence of demographic variables on depression among females with SUD using Student's *t*-test or analysis of variance (ANOVA). Next, Mplus 7.0 was used to test the hypothesized model. Because depression may be correlated with a variety of sociodemographic factors, the hypothesized model was conducted by adding age, marital status (married or not), education, duration of substance use, number of compulsory detoxifications, and type of substance used as control variables, which is a common statistical method to reduce the confounding effects of personal characteristics (68). A path analysis model was conducted to test the mediating roles of IU and anhedonia in the relationships between the BIS/BAS and depression in females with SUD. In the current study, several goodness-of-fit indices were adopted to test the model-data fit. The chi-square statistic and its associated *p*-value were reported. If the *p*-value is not significant, it may show good model-data fit. Other model fit indices include the Tucker-Lewis Index (TLI) (69), the comparative fit index (CFI) (70), the standardized root mean square residual (SRMR) (71) and the root mean square error of approximation (RMSEA) (72). A TLI and CFI greater than 0.95 and an SRMR and RMSEA <0.08 indicate good model fit (71). The bias-corrected percentile bootstrap method (5,000 bootstrap samples) with 95% confidence intervals (CIs) was performed to examine the significance of mediation effects. The 95% CIs that do not contain zero show that the effects are significant. The predictive and explanatory powers of the model were assessed using path coefficients and *R*^2^.

## Results

### Impact of Demographic Features on Depression

Briefly speaking, there were no significant differences in depression between two kinds of marital status including married or not (*t*_(296)_ = 1.60, *p* = 0.11), among education groups (*F*(3, 294) = 1.12, *p* = 0.34), among occupational status groups (*F*(3, 291) = 1.20, *p* = 0.31), among duration of substance use groups (*F*(4, 295) = 0.40, *p* = 0.81), and among number of compulsory detoxification groups (*F*(2, 294) = 0.04, *p* = 0.96). However, there was a significant difference in depression between the two types of substance used groups (*t*_(290)_ = 2.37, *p* = 0.02). Females with traditional substance (M ± SD = 17.37±9.46) had higher level of depression than others with new substance (M ± SD = 14.41 ± 8.85). In addition, according to a CES-D cutoff score of ≥16 indicating depressive symptoms, there were 137 (45.2%) depressed females in our sample.

### Descriptive Statistics and Correlations

The descriptions and correlations of all variables from these scales are presented in Table 2. Specifically, the BIS was positively associated with IU but negatively associated with anhedonia. The BAS was negatively correlated with anhedonia and depression. Both IU and anhedonia were positively associated with depression.

**Table 2:** Mean, standard deviation, and correlations among all variables.

	**M**	**SD**	**1**	**2**	**3**	**4**	**5**
1. BIS	15.73	2.45	1				
2. BAS	43.29	6.12	0.54^**^	1			
3. IU	78.79	15.62	0.29^**^	0.08	1		
4. Anhedonia	22.71	5.97	−0.22^**^	−0.40^**^	−0.09	1	
5. Depression	14.93	9.14	0.08	−0.20^**^	0.19^**^	0.28^**^	1

BIS, behavioral inhibition system; BAS, behavioral activation system; IU, intolerance of uncertainty;

** *p < 0.01*.

### Path Analysis Model

**Figure 2:**
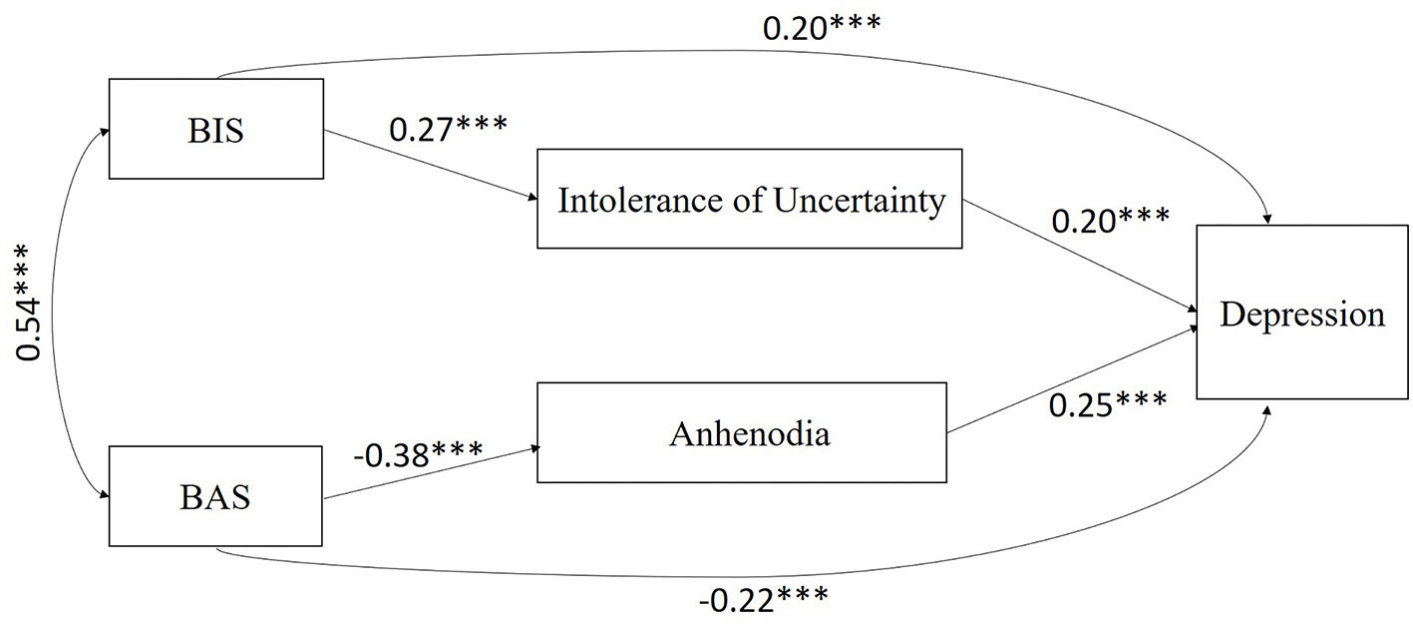
The relationships between BIS/BAS and depression mediated by intolerance of uncertainty and anhedonia. ***p < 0.001.

The results of the initial hypothesized model with age, marital status, education, duration of substance use, number of compulsory detoxifications, and type of substance used as control variables (χ2 = 36.810, χ2/df = 1.472, *p* = 0.060, TLI = 0.889, CFI = 0.897, SRMR = 0.040, RMSEA = 0.041) showed that the fit of the model was suboptimal, but there were eight non-significant pathways for age and depression (β = 0.008, *p* = 0.905), marital status and depression (β = −0.104, *p* = 0.056), education and depression (β = 0.046, *p* = 0.424), duration of substance use and depression (β = 0.093, *p* = 0.091), number of compulsory detoxification and depression (β = 0.016, *p* = 0.823), type of substance used and depression (β = −0.021, *p* = 0.748), the BIS and anhedonia (β = −0.042, *p* = 0.555), and the BAS and IU (β = −0.114, *p* = 0.076) in this model. After removing these eight pathways, the results of the measurement showed that the modified model fit the data excellently (χ^2^ = 3.922, χ^2^/df = 1.307, *p* = 0.270, TLI = 0.976, CFI = 0.992, SRMR = 0.021, RMSEA = 0.033). Standardized pathway coefficients within factors are displayed in Figure 2. The final model accounted for 16.6% of the total variance in depression among females with SUD.

When the final model was chosen, bias-corrected bootstrapping was performed to further test the significance of the mediators. Compared to traditional mediation analyses, bootstrapping as a non-parametric resampling procedure can provide greater statistical power to test indirect effects (73). The results of the bootstrap analyses indicated that the direct effect of the BIS on depression was significant (β = 0.201, *p* = 0.001, 95% CI = [0.080, 0.323]), and the specific indirect effect of the BIS on depression through IU was also significant (β = 0.054, *p* = 0.001, 95% CI = [0.021, 0.087]). In addition, the results of the bootstrap analyses showed that the direct effect of the BAS on depression was significant (β = −0.219, *p* < 0.001, 95% CI = [−0.336, −0.103]), and the specific indirect effect of the BAS on depression *via* anhedonia was also significant (β = −0.095, *p* < 0.001, 95% CI = [−0.148, −0.043]).

## Discussion

To the best of our knowledge, this is the first study to explore the direct and indirect effects of the BIS/BAS on depression through IU and anhedonia in females with SUD within the RDoC framework. The present results provide strong evidence supporting our proposed model. We found a high prevalence of depression in this population and that the BIS significantly positively predicted depression, while the BAS significantly negatively predicted depression. In addition, IU was a significant mediator between the BIS and depression, while anhedonia was a significant mediator between the BAS and depression in this population. Considering that SUD differ in course and outcome between women and men, the present results for females with SUD could contribute to understanding depression status, understanding the mechanism of depression, and providing important guidelines for the identification, intervention and treatment of depression in this special population.

In the current study, 45.2% of the females with SUD reported having depression, which is much higher than the rates in other populations (e.g., general women, male with SUD) in previous studies (15, 74). Several reasons may explain why there was a high prevalence rate found in the present study. First, depression is one of the most common psychiatric disorders and a major public health problem in the Chinese population, especially among women, because great social and economic transformation, as well as rapid urbanization and modernization in China, brought dramatic changes to society, including increased personal and contextual stressors (i.e., faster pace of life, job loss, marital divorce or separation, traffic congestion, overcrowded living conditions), disintegration of China's traditional large family, and decline in family support by weakening family ties (75). Most of these factors have been considered risk factors for depression in a previous study (76) and may correlate with the increased depression prevalence rate found in females with SUD. In the present study, 78.2% of the sample was unmarried or divorced, which was likely to cause difficulties in the availability of family support, one of the most important sources of social support in Chinese culture. Second, compared to general women, females with SUD may experience more stressful events, such as underemployment, broad socioeconomic disadvantage and poor health status, which are potential risk factors for depression. Third, compared to male with SUD, substances could have more serious negative consequences in females with SUD. These consequences include higher substance use dependence, easier relapse, less response to treatment, more severe SUD syndrome, and higher comorbidity of SUD and depression (14, 15).

Compared to the positive predictors of high BIS and BAS levels for SUD found in a previous study (24, 25), the current results indicated that high BIS levels and low BAS levels directly positively predict depression in females with SUD, which is supported by the theory that both avoidance motivation and approach deficits play an important role in weakening positive experiences and reinforcement for non-depressed behaviors, leading to the onset and maintenance of depression (11). On the one hand, individuals with reward hypersensitivity are more likely to be susceptible to SUD by means of substance-triggered excessive reward activation states. When their reward systems switch to excessively deactivate responsivity to unresolved failures or losses because of the termination of substance use in the compulsory substance abuse detention center, they become more vulnerable to depression, which is supported by electrophysiological studies (30). For example, a previous study found that low BAS levels measured by ERPs prospectively predict depression onset in adolescent girls (77). Furthermore, a longitudinal fMRI reward process study has indicated that compared with healthy controls, individuals with depression who consistently exhibited less striatal activation to reward stimuli suffered from increasing depressive symptoms (78). On the other hand, females with high BIS levels, who are more likely to show hypersensitivity to punishment stimuli (e.g., failure or loss, criticism or scolding), are more vulnerable to substance overuse to escape loneliness and life problems. Meanwhile, females with SUD with high BIS levels have been shown to be prone to depression because the BIS is positively correlated with neuroticism, which is a risk personality trait for depression (79).

In the current study, we found that IU mediated the relationships between the BIS and depression and that anhedonia served as a mediator of the relationships between the BAS and depression among females with SUD, which shows that these variables serve as indicators of differential risk factors with great importance to support the unique mechanisms corresponding to depressive symptoms in females with SUD within the RDoC framework (19). On the one hand, the BIS can intensify individual reactions to withdraw from or avoid novel, uncertain or threatening contexts, which may trigger individuals to interpret ambiguous information more negatively (41). Especially for females with SUD who suffer from more negative life events and more uncertainty within their environment, high BIS levels could induce high IU, thus leading to a higher level of depression than the individuals with low BIS levels. Because individuals with high levels of IU show blunted reward responsiveness (40), the BAS cannot have an indirect impact on depression through IU. On the other hand, the BAS has a close relationship with reward seeking, and individuals with high BAS sensitivity show a high preference for novelty processing and reward dependence (23). The BAS may play an important role in individuals' motivations to conduct goal-directed behavior (52). However, individuals with a high level of anhedonia take part in less pleasant activities on the non-substance-related activities list and less physical activities (e.g., walking frequency, moderate-intensity physical activity frequency and duration, and vigorous-intensity physical activities and duration), which are associated with higher physical activity enjoyment and a lower level of depressive symptoms (51, 52). Thus, females with SUD with low BAS levels lose their motivation to pursue meaningful and rewarding stimuli and experience more anhedonia in daily life, which exacerbates their depression. However, individuals with high BIS sensitivity show an enhanced preference for punishment and threat stimuli rather than pleasure or reward stimuli (23), which could explain why the BIS did not indirectly influence depression via anhedonia in females with SUD in the present study.

The present study may have some theoretical and practical implications. From a theoretical perspective, these results provide strong evidence in support of the RDoC initiative. First, consistent with previous findings (29, 46, 47, 55), the present study found that the BIS, the BAS, IU, and anhedonia have close relationships with depression in females with SUD, which indicates that these variables are transdiagnostic features across different psychiatric disorders. Second, the BIS had an indirect influence on depression through IU, while the BAS had an indirect influence on depression *via* anhedonia in the present study, which suggests that it is imperative to explore the specific mechanisms unique to specific psychiatric symptoms (19). From a practical perspective, the current results could aid evidence-based prevention and interventions to decrease depression among females with SUD. Based on our model, prevention and interventions considering the BIS/BAS, IU, and anhedonia may be helpful for establishing effective strategies for females with SUD with depression. First, when identifying a target population for further prevention and intervention programs among females with SUD, combinations of these risk factors (e.g., low BAS levels, high BIS levels, high IU, and high anhedonia) should be adopted according to the present results. Second, and even more importantly, our results provide invaluable knowledge on how to prevent and intervene in depression among females with SUD. Specifically, interventions to decrease BIS levels and to increase BAS levels could decrease depression in females with SUD. In addition, the findings of this study that IU mediates the associations between the BIS and depression and that anhedonia mediate the relationships between the BAS and depression has important implications for practice. On the one hand, to prevent and intervene in depression in females with SUD with high BIS levels, CBT-IU techniques should be exploited to enhance females with SUD' acceptance of uncertainty because these techniques include psychoeducation on reappraising uncertainty, cognitive modifications for unrealistically positive illusions about seeking certainty, and exposure training for uncertainty (37). On the other hand, to prevent and intervene in depression in females with SUD with low BAS levels, specific behavioral activation therapy unique to these populations should be given more attention and deserve further exploration (57).

However, there are some limitations in the present study that have to be addressed. First, there was the absence of structured assessment data to validate the clinical diagnoses of depression in the sample, although the CES-D has been widely used to assess depression in epidemiological research (75). Thus, future studies should try to assess depression in females with SUD based on a clinical diagnosis system (e.g., DSM-V or ICD-11). Second, this study was a cross-sectional survey study, which prevented the identification of any causal relations among these variables. Future studies should conduct experimental research or use longitudinal designs to strictly explore the causal relationships among these variables. Third, this study adopted the self-report method, which may restrict the validity of the data because of social desirability and memory or response biases. Therefore, future studies should take efforts to collect data from multiple informants (e.g., females with SUD, supervisors and peers) and use multi-index methods, including objective markers and subjective reports. Fourth, the physical health status of the sample was not assessed in the present study, that may ignore the potential influence of physical health on the present results. Therefore, future studies should try to assess physical health status in females with SUD. Finally, although the results from removing the subjects with missing data and redoing the analysis were the same as the present results, some potential errors may appear through the methods that some missing data were replaced with corresponding mean values and then were included in the analysis. Thus, future studies may increase sample size and improve their survey response quality to provide more strong evidence supporting the present findings.

## Data Availability Statement

The raw data supporting the conclusions of this article will be made available by the authors, without undue reservation.

## Ethics Statement

The studies involving human participants were reviewed and approved by the ethics committee of Department of Psychology, Capital Normal University. The patients/participants provided their written informed consent to participate in this study.

## Author Contributions

JX and PF designed the study and wrote the protocol. BD and ZZ collected the research data. JX and BD conducted the statistical analyses and wrote the manuscript. RL conducted the literature searches and created the figures. All authors approved the final version of the manuscript.

## Conflict of Interest

The authors declare that the research was conducted in the absence of any commercial or financial relationships that could be construed as a potential conflict of interest.
